# Supplementary material for: Socioeconomic status and subjective well-being: The mediating role of class identity and social activities
Source: PLoS One. 2023 Sep 15;18(9):e0291325. doi: 10.1371/journal.pone.0291325 (PMC10503752; doi:10.1371/journal.pone.0291325)
Supplement: Attachment — Submitted filename: Response to Reviewers.docx [file pone.0291325.s002.docx]

Dear Editor,

We appreciate your reply. We apologize for the incorrect formatting of the references due to our mistake. We have adjusted the reference format to ensure it is complete and correct. And all of the cited literature is published. Appended to this letter is our point-by-point response to the comments raised by the reviewers. Changes are shown in red in the revised version.

Kind regards,

Yi Jiang

**Responds to the reviewers' comments**

**Reviewer 2**

1 Based on the changes made to the Manuscript by the authors, it appears that the text has undergone an acceptable upgrade. Therefore, there is no more comment in this regard.

Response: Thank you very much for your valuable suggestions and comments.

**Reviewer 3**

1 Thank you for your previous detailed responses to my comments. While I appreciate the explanation to point one, it is still unclear to a reader what those numbers represent. It is not immediately clear in the abstract and results that this was a multivariate linear regression analysis. The authors can consider reporting as: "For each unit increase in socioeconomic status score, the subjective well-being score was expected to increase by 0.351 (95% CI 0.721, 1.587)..."

Response: We appreciate your comments very much. We apologize for not clearly showing that the chain mediation analysis is based on several multiple linear regressions (as shown in Table 3). We have adapted pertinent elucidations within the manuscript to explicitly expound that the chain-mediated model comprises the four multiple linear regressions presented in Table 3. Furthermore, we utilized Figure 2 to present the connection between socioeconomic status, class identity, participation in social activities, and subjective well-being more visually. Regarding the query that the various coefficients (β) and p values we report may be difficult for the reader to understand, we inquired about how these coefficients are reported in the relevant literature. We found that the presentation of the chain-mediated modeling results in the manuscript is consistent with other relevant literature (DOI numbers are displayed at the end), expressed as the effect of A on B (coefficients and p-values, or 95% CI). This mode of presentation indicates the direction, degree, and precision of the connection between the two-by-two variables, which may not affect the reader's understanding of the results. The reader can interpret the coefficients (β) and p-values as reported by the multiple linear regression. For example, "socioeconomic status was a significant predictor of individuals' subjective well-being (β = 1.583, p < 0.01)" can be interpreted as "for every one-unit increase in socioeconomic status score, the subjective well-being score was expected to increase by 1.583 (p < 0.01)." Moreover, we believe that this presentation is concise and clear, thereby reducing the reading burden on the reader.

The various indirect effects are products of the corresponding path coefficients. The magnitude of the indirect effect indicates how much of the total effect between the independent variable (X) and the dependent variable (Y) is explained by the mediating variable (M). The significance, along with the 95% confidence interval, of an indirect effect determines whether the observed indirect effect may have occurred at random. Furthermore, 95% confidence intervals for indirect, direct, and total effects were tested using the Bootstrap method to ensure that these findings were not coincidental (as shown in Table 4). Therefore, the interpretation of the magnitude of the indirect effect may differ from the preceding regression coefficients.

The details of the modifications are shown as follows:

The analysis of the chain mediation model included four multiple linear regressions, each accounting for covariates. Table 3 and Figure 2 present the results of the chain mediation analysis for this study. In the total effects model, SES was a significant predictor of individuals' SWB (β = 1.583, p < 0.01). This relationship remained significant when class identity and social activity were also considered (β = 0.841, p < 0.05). Furthermore, SES has a significant effect on class identity (β = 0.289, p < 0.01) and social activity (β = 2.699, p < 0.01). Class identity and social activity were also significant predictors of SWB, with corresponding coefficients of 1.213 (p < 0.01) and 0.141 (p < 0.01). Class identity was a significant predictor of social activity, with a coefficient of 0.274 (p < 0.05). Using these path coefficients, the corresponding indirect effects were calculated. The indirect effects of SES on SWB, mediated by class identity and social activity, were 0.351 and 0.380, respectively. The chain mediation effect was 0.011, indicating that the effect of SES on SWB was transmitted between the mediating variables. (Line 209-222)

The DOI numbers of the references for the reporting style are specified below:

(1) 10.1371/journal.pone.0122128

(2) 10.1371/journal.pone.0231628

(3) [10.1371/journal.pone.0280701](https://doi.org/10.1371/journal.pone.0280701)

(4) 10.1186/s12889-022-14711-7

(5) [10.1371/journal.pone.0289092](https://doi.org/10.1371/journal.pone.0289092)
